# Multiple cardiometabolic diseases enhance the adverse effects of hypoalbuminemia on mortality among centenarians in China: a cohort study

**DOI:** 10.1186/s13098-023-01201-y

**Published:** 2023-11-14

**Authors:** Haowei Li, Shengshu Wang, Shanshan Yang, Shaohua Liu, Yang Song, Shimin Chen, Xuehang Li, Zhiqiang Li, Rongrong Li, Yali Zhao, Qiao Zhu, Chaoxue Ning, Miao Liu, Yao He

**Affiliations:** 1 Institute of Geriatrics, Beijing Key Laboratory of Aging and Geriatrics, National Clinical Research Center for Geriatrics Diseases, Second Medical Center of Chinese, PLA General Hospital, 28 Fuxing Road, Beijing, 100853 China; 2 Department of Healthcare, Agency for Offices Administration, Central Military Commission, People’s Republic of China, Beijing, 100082 China; 3 Department of Disease Prevention and Control, Chinese PLA General Hospital, The 1St Medical Center, Beijing, 100853 China; 4 Special Combat Detachment of Xinjiang Armed Police Crops, Health Corps, Aksu, 843000 China; 5 Chinese PLA Center for Disease Control and Prevention, Beijing, 100071 China; 6 Central Laboratory of Hainan Hospital, Chinese PLA General Hospital, Sanya, 572013 China; 7 Department of anti-NBC Medicine, Graduate School of Chinese PLA General Hospital, 28 Fuxing Road, Beijing, 100853 China; 8 State Key Laboratory of Kidney Diseases, Chinese PLA General Hospital, 100853 Beijing, China

**Keywords:** Serum albumin, Cardiometabolic diseases, All-cause mortality, Centenarians, Cohort study

## Abstract

**Background:**

Although hypoalbuminemia was associated with high risk of mortality in community-dwelling older adults, as well as in the hospitalized older adults, little is known among centenarians. And there are limited data on whether having cardiometabolic diseases (CMDs) is associated with additive effects.

**Methods:**

Baseline examinations including a determination of albumin levels were performed in 1002 Chinese centenarians from January 2014 through to December 2016, and the survival status was subsequently ascertained until 31 May 2021. Cox proportional risk model was performed to assess the risk of all-cause mortality associated with albumin levels and hypoalbuminemia combined with CMDs.

**Results:**

Of 1002 participants included in the analysis, the mean level of albumin was 38.5 g/L (± standard deviation, 4.0 g/L), and 174 (17.4%) had hypoalbuminemia (albumin < 35 g/L). The multivariable analyses showed that albumin level was negatively associated with all-cause mortality (*P*_trend_ < 0.05). Compared to normoalbuminemia, hypoalbuminemia was associated with an increased mortality risk in the overall participants (hazard ratio [HR]: 1.55, 95% confidence interval [CI]: 1.22–1.97). Furthermore, the HR (95% CI) of hypoalbuminemia combined with multiple CMDs was 2.15 (1.14–4.07). There was evidence of an additive deleterious dose effect of an increasing number of CMDs (*P*_trend_ = 0.001).

**Conclusions:**

Hypoalbuminemia is associated with an increased risk of all-cause mortality in Chinese centenarians, and this risk is more pronounced among centenarians with multiple cardiometabolic diseases. Our findings suggest that older adults with hypoalbuminemia, especially comorbid multiple CMDs warrant early identification and management.

**Supplementary Information:**

The online version contains supplementary material available at 10.1186/s13098-023-01201-y.

## Background

Serum albumin is one of the earliest recognized and abundant proteins, with its synthesis accounting for approximately a quarter of total hepatic protein synthesis [1]. Since albumin provides a relatively objective reflection of the body's protein reserve levels, it has been widely recognized as an evaluation tool for nutritional status [2]. Low albumin level has been used as a marker of malnutrition related to increased risk of adverse health outcomes, including death and other severe diseases [3–8]. Besides the association between low serum albumin levels and malnutrition, these findings have additionally been supported by the decline in the function of albumin as an extracellular antioxidant agent [9–11]. Moreover, albumin is the principal negative acute-phase reactant, and its level falls in response to inflammation during acute critical illnesses or chronic systemic diseases, making a decrease in albumin an indicator of increased risk of adverse outcomes [12–14].

Many studies have reported that hypoalbuminemia is associated with high mortality in patients with various diseases or in healthy community-dwelling older adults [5, 15–18]. However, most of the current older adults, especially those of advanced aged, exist with cardiometabolic diseases (CMDs) including diabetes, coronary heart diseases (CHD), stroke and hypertension [19–21]. Of interest is that inflammation and nutritional problems are present in older adults with CMDs, while whether CMDs has an impact on the association between albumin, a dual indicator of inflammation and nutrition, and mortality is not clear. Prior studies have suggested that the association between lower albumin and increased risk of death in patients with acute coronary syndrome, stroke and acute heart failure [16, 22–25]. However, presently, there is a lack of large-scale research on the potential effect of increasing number of CMDs on the association between hypoalbuminemia and mortality, especially among Chinese centenarians.

Therefore, this study attempts to detect the relationship between serum albumin levels and the risk of all-cause death, and to explore the additional effects of CMDs on such an association in the community-based centenarians, using data from the China Hainan Centenarian Cohort Study (CHCCS), to provide ideas for the early identification and intervention of hypoalbuminemia in the centenarians.

## Methods

### Study design and participants

Study participants were recruited from the CHCCS Project, a longevity population-based study. A detailed description of study design framework has previously been published [26]. This prospective cohort study included 1,002 eligible centenarians aged more than 100 years old residing in 16 cities and counties of Hainan province from January 2014 through to December 2016. Participants were subsequently contacted until 31 May 2021 to obtain follow-up information including death for any cause.

This study was approved by the Biomedical Ethics Committee of Chinese PLA general hospital (301hn11-206-01), and was performed in accordance with the Declaration of Helsinki. All participants gave written informed consent.

### Investigation method

We had a face-to-face interview with each study subject in the participants’ residences or nearby clinics through appropriate regional dialects. Demographic and sociological characteristics, personal disease and family histories, lifestyle and disability were collected by a standard questionnaire. All the questions on the questionnaire were asked and recorded by trained investigators from Hainan Hospital affiliated to the Chinese PLA General Hospital. Anthropometric measurements and blood pressure levels were recorded during physical examination. Body mass index is calculated as weight in kilograms divided by height squared in meters (For those with marijuana, measure their length instead of height). Blood samples were collected after a 8-h overnight fasting by venous puncture and sent to the department of biochemistry, Hainan Hospital, within 4 h, to test the level of total cholesterol (TC), triglyceride (TG), high density lipoprotein cholesterol (HDL-C), low density lipoprotein cholesterol (LDL-C), fasting blood glucose (FBG), total protein, albumin, aspartate aminotransferase (AST), alanine transaminase (ALT), hemoglobin, CRP and creatinine.

### Measurement and classification of albumin

Comprehensive metabolic panel examinations were determined on Cobas 8000 automatic biochemical autoanalyzer (Roche Products Ltd., Basel, Switzerland). Albumin levels was divided into quartiles: < 36.2 g/L, 36.2–38.9 g/L, 39.0–41.2 g/L, and ≥ 41.3 g/L, and further dichotomized: more than 35 g/L and less than 35 g/L. Hypoalbuminemia is defined by a serum albumin < 35 g/L [27].

### Assessment of cardiometabolic diseases

In the present analysis, CMDs included diabetes, CHD, stroke and hypertension. As part of the baseline assessment, participants were asked whether a physician had diagnosed diabetes, CHD, stroke or hypertension. We defined hypertension as previous diagnosis or SBP ≥ 140 mmHg or DBP ≥ 90 mmHg [28], and diabetes as previous diagnosis or FBG ≥ 7.0 mmol/L [29]. CHD and stroke history was finally determined from their medical records output from Grade II and III hospitals. The CMDs status was categorized as CMD-free, single CMD (any one of the above CMDs) and multiple CMDs (two or more the above of CMDs).

### Outcomes

The death information was ascertained through linkage to the death registration system of the public security department and the registration system of the Hainan Provincial Committee on Aging, and was verified by telephone. After the survival status was confirmed, the study came to an end. There was no missing follow-up until May 31, 2021.

### Covariates

Socio-demographic characteristics assessed included age, sex, education, ethnicity and marriage. The ethnicity of a subject is classified as either Han or minority. Marital status is divided as widowed or not (including married, divorced, or other). The education level is classified as illiteracy or not according to the total years of education. Smoking status, alcohol drinking status and disability assessment were also collected at the same time. Smoking status and alcohol drinking status are classified into two categories: current or former, and never. Dyslipidemia was defined as previous diagnosis or TC ≥ 6.22 mmol/L, or TG ≥ 2.26 mmol/L, or HDL-C < 1.04 mmol/L, or LDL-C ≥ 4.14 mmol/L [30]. Activities of daily living (ADL) function was evaluated using the Barthel index [31], which evaluates 10 different instrumental ADL with a maximum score of 100. The ADL score was categorized into two groups to reflect degrees of ADL disability: 1) ADL independent (ADL score > 40); 2) Having an ADL disability (ADL score ≤ 40). Estimated glomerular filtration rate (eGFR) was calculated by CKD epidemiology collaboration (CKD-EPI) [32].

### Statistical analysis

Continuous variables, expressed as mean (± standard deviation) in case of normally distributed variables or as median (interquartile ranges), were compared using the unpaired Student’s t-test, Mann–Whitney U-test, or One-way analysis of variance, as appropriate. Categorical variables, expressed as count (percent), were compared using the chi-square test. We used multivariable adjusted restricted cubic splines to examine possible albumin threshold levels for mortality, as well as the potential non-linear association with the risk of all-cause death. Time data for the occurrence of death events were graphically presented using the Kaplan–Meier method. After verification of proportionality assumptions, the univariate and multivariable Cox proportional risk model was used to calculate the hazard ratios (HRs) and 95% confidence intervals (CIs) of follow-up all-cause mortality associated with albumin levels and hypoalbuminemia combined with different numbers of CMDs. The models were adjusted for sex, age, ethnicity, marriage, educational levels, smoking status, alcohol drinking status, dyslipidemia, diabetes, CHD, stroke, hypertension, hemoglobin and CRP. Sensitivity analyses were conducted based on the following in further analyses to test the robustness of the primary results: a. Given to some studies add dyslipidemia in the definition of CMDs [33], we performed sensitivity analyses including dyslipidemia as a component for the definition of CMDs. b. The centenarians with ADL independent may nonetheless have higher levels of total daily physical activity compared to the centenarians with ADL disability, who might be poorer in nutritional status including hypoalbuminemia. Thus, those with ADL disability were excluded to minimize the possibility of reverse causation bias by baseline health status. c. Additional adjustment for eGFR to reduce the confounding effect of renal excretion on the relationship between albumin levels and mortality. Statistical analyses were performed using SPSS Statistics version 24.0 (IBM Corporation, Armonk, NY, United States) and R version 3.6.0 (R Foundation for Statistical Computing). Two-tailed *p* values of < 0.05 were considered statistically significant.

## Results

### Baseline characteristics

A total of 1002 participants were enrolled in our analysis, with 82.0% (822) females and median aged 102 (101–104) years old, and baseline characteristics were shown in Table 1 and Additional file 1: Table S1. Among centenarians, there was a high percentage of Han ethnicity, widowed and illiterate. The proportions of participants who never smoking and alcohol drinking were all higher than 80%. The centenarians who died had lower levels of albumin and higher prevalence of hypoalbuminemia. Albumin had a normal distribution with mean (± standard deviation) values of 38.5 g/L (± 4.0 g/L) and ranged from 23.1 to 52.2 g/L. The prevalence of hypoalbuminemia for our study was 17.4%. The distribution of albumin was depicted in Additional file 1: Figure S1. In addition, the prevalence of hypertension, diabetes, CHD and stroke were 74.7%, 9.6% 4.1% and 2.2%, respectively. The participants with lower albumin levels were tend to have significantly higher CRP levels and prevalence of ADL disability, while lower BMI, blood pressure, total protein levels and hemoglobin levels (all *p* < 0.05).

**Table 1:** Baseline characteristics of the study participants

	Total	Serum albumin				*P* value
		Q4 (≥ 41.3 g/L)	Q3 (38.9–41.2 g/L)	Q2 (36.2–38.9 g/L)	Q1 (< 36.2 g/L)	
Age(year), median(IQR)	102 (101–104)	102 (101–104)	102 (101–105)	102 (101–105)	102 (101–105)	0.026
Female, n(%)	822 (82.0)	203 (84.2)	213 (83.5)	197 (79.1)	209 (81.3)	0.440
Han ethnicity, n(%)	883 (88.1)	213 (88.4)	217 (85.1)	225 (90.4)	228 (88.7)	0.318
Widowed, n(%)	836 (83.4)	199 (82.6)	220 (86.3)	208 (83.5)	209 (81.3)	0.485
Illiterate, n(%)	915 (91.3)	217 (90.0)	236 (92.5)	223 (89.6)	239 (93.0)	0.762
Never smoking, n(%)	896 (89.4)	215 (89.2)	232 (91.0)	223 (89.6)	226 (87.9)	0.736
Never alcohol drinking, n(%)	836 (83.4)	201 (83.4)	217 (85.1)	212 (85.1)	206 (80.2)	0.386
ADL disability, n(%)	140 (14.0)	7 (2.9)	29 (11.4)	26 (10.4)	78 (30.4)	< 0.001
Hypertension, n(%)	744 (74.3)	202 (83.8)	199 (78.0)	176 (70.7)	167 (65.0)	< 0.001
Diabetes, n(%)	96 (9.6)	29 (12.0)	17 (6.7)	23 (9.2)	27 (10.5)	0.216
CHD, n(%)	41 (4.1)	13 (5.4)	11 (4.3)	10 (4.0)	7 (2.7)	0.512
Stroke, n(%)	22 (2.2)	5 (2.1)	7 (2.7)	9 (3.6)	1 (0.4)	0.085
Dyslipidemia, n(%)	230 (23.0)	59 (24.5)	52 (20.4)	55 (22.1)	64(24.9)	< 0.001
BMI(kg/m^2^), mean ± SD	18.04 ± 3.41	18.46 ± 3.24	18.13 ± 3.25	18.27 ± 3.20	17.33 ± 3.79	0.001
SBP(mmHg), mean ± SD	152.57 ± 24.43	159.90 ± 24.85	154.99 ± 22.46	151.51 ± 24.59	144.31 ± 23.29	< 0.001
DBP(mmHg), mean ± SD	75.75 ± 12.94	79.99 ± 14.02	77.09 ± 11.37	74.62 ± 12.13	71.53 ± 12.72	0.001
CRP (mg/L), median (IQR)	0.19 (0.08–0.43)	0.13 (0.05–0.30)	0.19 (0.09–0.33)	0.17 (0.07–0.40)	0.33 (0.14–0.99)	< 0.001
Haemoglobin (g/L), mean ± SD	113.05 ± 16.35	120.73 ± 15.44	115.73 ± 13.69	112.17 ± 15.30	104.04 ± 16.27	< 0.001
FBG(mmol/L), median(IQR)	4.82 (4.26–5.64)	4.82 (4.20–5.25)	4.82 (4.32–5.62)	4.82 (4.23–5.67)	4.97 (4.27–5.85)	0.225
eGFR(mL/min/1.73 m^2^), median (IQR)	76.42 (48.62–100.96)	80.87 (47.99–103.46)	76.42 (49.82–97.74)	71.52 (46.88–99.78)	77.72 (49.32–101.18)	0.724
Total protein (g/L), mean ± SD	68.65 ± 6.27	73.00 ± 5.88	69.65 ± 5.18	67.62 ± 4.90	64.73 ± 5.87	< 0.001
ALT (UI/L), median(IQR)	9.20 (7.20–12.00)	10.00 (8.20–12.95)	9.40 (7.70–11.80)	8.50 (7.10–12.35)	8.20 (6.40–10.85)	0.086
AST (UI/L), median(IQR)	20.60 (17.50–24.40)	21.50 (18.90–25.65)	20.70 (17.95–24.60)	20.20 (17.40–23.60)	19.40 (16.60–23.90)	0.396

*ADL* activities of daily living, *ALT* alanine transaminase, *AST* aspartate aminotransferase, *BMI* body mass index, *CHD* coronary heart diseases, *CRP* C-reactive protein, *DBP* diastolic blood pressure, *eGFR* estimated glomerular filtration rate, *FBG* fasting blood glucose, *HDL* high-density lipoprotein, *IQR* interquartile ranges, *LDL* low-density lipoprotein, *SBP* systolic blood pressure, *SD* standard deviation, *TC* total cholesterol, *TG* triglycerides

### Association between albumin levels and all-cause mortality

**Fig. 1:**
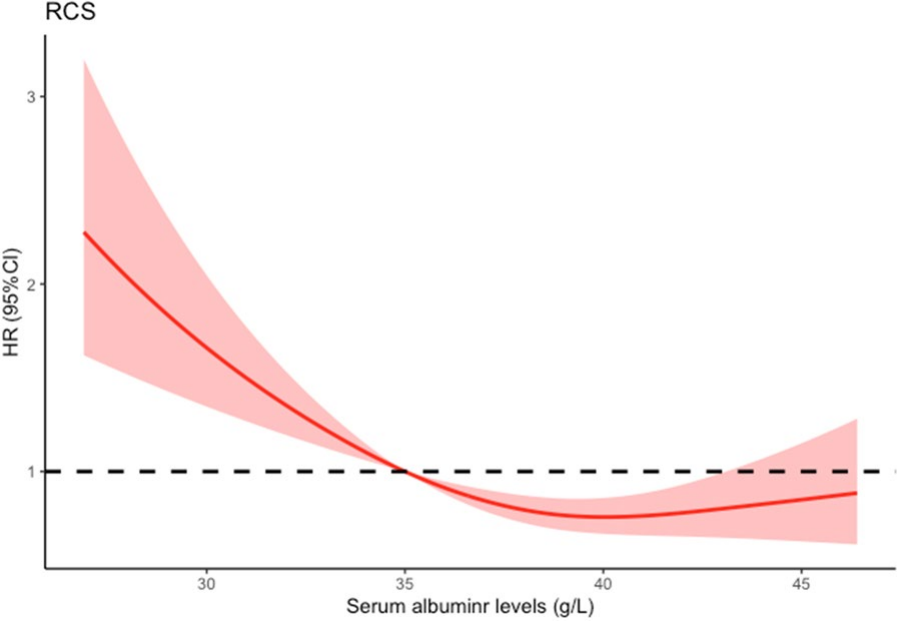
Restricted cubic spline of the association between albumin levels and risk of all-cause death. Adjusted for sex, age, ethnicity, marriage, educational levels, smoking status, alcohol drinking status, dyslipidemia, hypertension, diabetes, CHD, stroke, hemoglobin and CRP. *CHD* coronary heart diseases, *CI* confidence interval, *CRP* C-reactive protein, *HR* hazard ratio

During the median follow-up time of 49.9 months, 522 (52.1%) all-cause deaths were observed. When treating albumin as a continuous variable, the restricted cubic spline analysis showed a flat L-shape with the inflection point at about 35 g/L (Fig. 1). A strong linear relationship was observed between a gradual rise in all-cause mortality risk and declining albumin levels, when the albumin level was < 35 g/L. However, when the albumin level was above the inflection point, the risk decreased and eventually leveled-off.

Compared with the highest quartile of albumin levels, the all-cause mortality was higher in participants with lower albumin levels (Additional file 1: Figure S2), and the multivariable-adjusted HRs for mortality in the third, second, and first quartile were 1.19 (95% CI: 0.92–1.53), 1.10 (0.85–1.44) and 1.45 (1.10–1.90) respectively, with a upward trend (*P*_trend_ = 0.021) (Table 2). In the dichotomous analysis of serum albumin using 35 g/L as the cutpoint, the cumulative incidence of death was 59.8% for participants with hypoalbuminemia and 50.5% for those with normoalbuminemia of ≥ 35 g/L, with significant differences shown in Kaplan–Meier curves (Additional file 1: Figue S3). In addition, hypoalbuminemia was associated with higher risk of all-cause mortality after multivariable adjustment (HR 1.55; 95% CI: 1.22–1.97) (Table 2).

**Table 2:** HRs and 95%CI of risks of all-cause death associated with albumin levels

Valid	Mortality	HR (95% CI)			
		Model 1	Model 2	Model 3	Model 4
Quintile variable					
Q1(< 36.2 g/L)	145 (56.4%)	1.38 (1.08–1.76)	1.38 (1.08–1.77)	1.40 (1.09–1.81)	1.45 (1.10–1.90)
Q2(36.2–38.9 g/L)	124 (49.8%)	1.09 (0.85–1.41)	1.08 (0.83–1.39)	1.09 (0.84–1.40)	1.10 (0.85–1.44)
Q3(39.0–41.2 g/L)	138 (54.1%)	1.20 (0.94–1.54)	1.18 (0.91–1.52)	1.17 (0.91–1.50)	1.19 (0.92–1.53)
Q4(≥ 41.3 g/L)	115 (47.7%)	1	1	1	1
*P* for trend	0.193	0.028	0.030	0.020	0.021
Binary variable					
Normoalbuminemia (≥ 35 g/L)	418 (50.5%)	1	1	1	1
Hypoalbuminemia (< 35 g/L)	104 (59.8%)	1.41 (1.14–1.75)	1.41 (1.14–1.75)	1.49 (1.20–1.86)	1.55 (1.22–1.97)

*CHD* coronary heart diseases, *CI* confidence interval, *CRP* C-reactive protein, *HR* hazard ratio

Model 1: No adjustment for any covariates

Model 2: Adjusted for sex, age, ethnicity, marriage, educational levels

Model 3: Model 2 plus smoking status, alcohol drinking status, dyslipidemia, hypertension, diabetes, CHD and stroke

Model 4: Model 3 plus hemoglobin, CRP

### Risk of mortality associated with hypoalbuminemia combined with CMDs

**Fig. 2:**
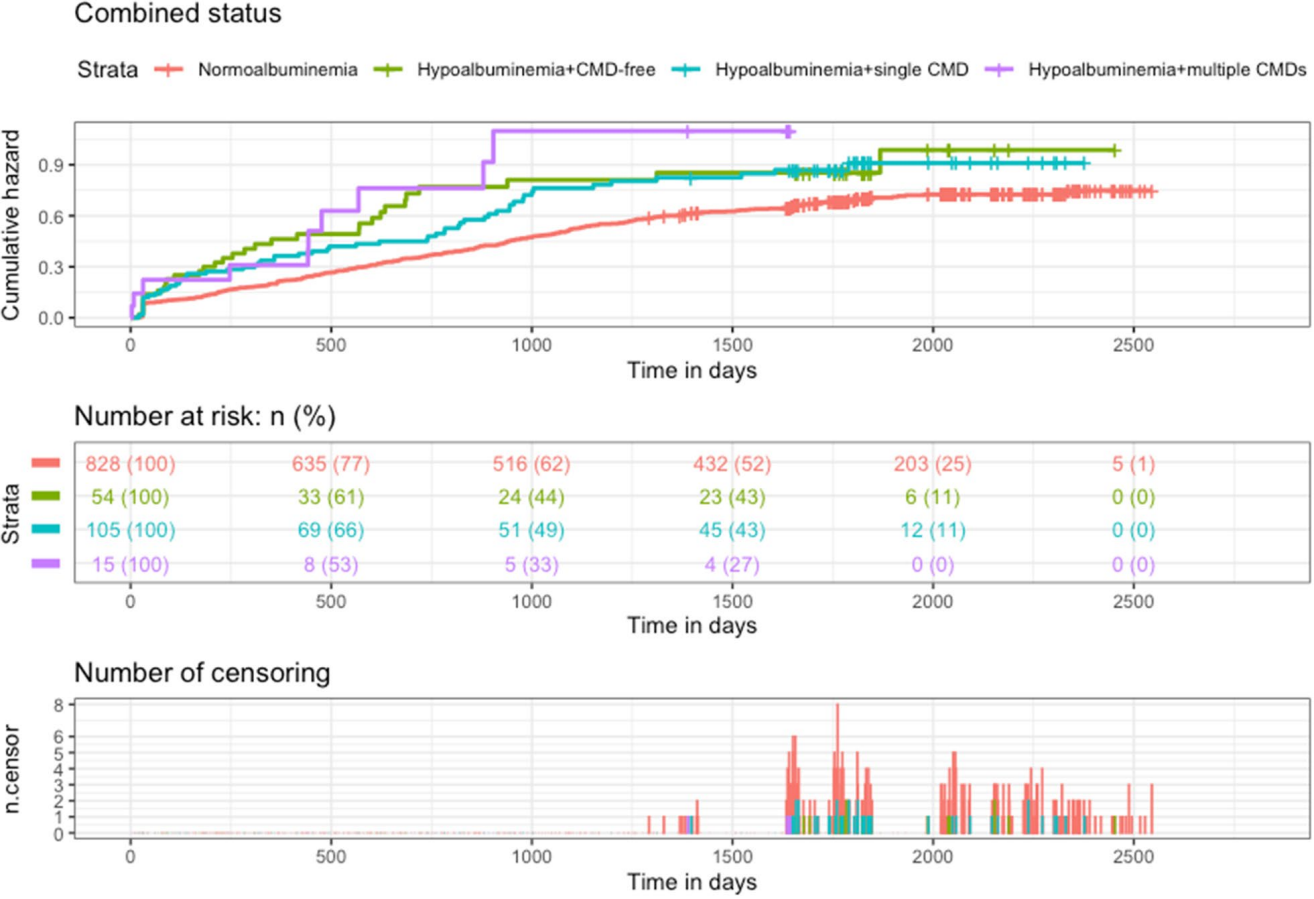
Kaplan–Meier curves for all-cause death by hypoalbuminemia combined with cardiometabolic diseases

The mortality risk for hypoalbuminemia were further analyzed taking into account the number of combined CMDs (Table 3). Among participants with hypoalbuminemia, 60.3% had single CMD and 8.6% had multiple CMDs. Compared to those with albumin levels ≥ 35 g/L, the cumulative mortality for participants with hypoalbuminemia combined with single CMD and multiple CMDs were noticeably higher (Fig. 2). The adjusted HRs were 1.45 (95%CI 1.08–1.93) and 2.15 (95%CI 1.14–4.07) respectively, and the increasing trend was significant (*P*_trend_ = 0.001). However, the number of CMDs itself was not related to a significantly increased risk of all-cause mortality (Additional file 1: Figure S4). Considering that a large proportion of centenarians with dyslipidemia, we preformed the analyses again after the inclusion of dyslipidemia into CMDs. The results were found to be similar (Additional file 1: Table S2).

**Table 3:** Risks of all-cause death associated with hypoalbuminemia combined with cardiometabolic diseases

Adverse outcome	Normoalbuminemia	Hypoalbuminemia combined with cardiometabolic diseases			
		0	1	≥ 2	*P* for trend
Mortality	418 (50.5%)	32 (59.4%)	62 (59.0%)	10 (66.7%)	0.152
HR (95% CI)					
Model 1	1	1.44 (1.01–2.06)	1.34 (1.03–1.75)	1.86 (0.99–3.48)	0.002
Model 2	1	1.44 (1.00–2.08)	1.34 (1.02–1.75)	1.97 (1.04–3.71)	0.002
Model 3	1	1.48 (1.02–2.13)	1.39 (1.06–1.81)	2.09 (1.11–3.93)	0.001
Model 4	1	1.54 (1.05–2.25)	1.45 (1.08–1.93)	2.15 (1.14–4.07)	0.001

*CI* confidence interval, *CRP* C-reactive protein, *HR* hazard ratio

Model 1: No adjustment for any covariates

Model 2: Adjusted for sex, age, ethnicity, marriage, educational levels

Model 3: Model 2 plus smoking status, alcohol drinking status, dyslipidemia

Model 4: Model 3 plus hemoglobin, CRP

Moreover, similar relationships were found after excluding participants with ADL disability or additionally adjusting for eGFR in the model, although some of the results were borderline significant due to smaller sample size (Additional file 1: Table S3, S4).

## Discussion

In this prospective cohort study of 1,002 Chinese centenarians with a median age of 102 years, hypoalbuminemia was frequently observed in centenarians with the prevalence of 17.4%, and was independently associated with increased all-cause mortality risk. Of note, the significant association was strongest with almost a doubling of risk in participants with comorbid hypoalbuminemia and multiple CMDs.

Significant associations between all-cause mortality and the lower serum albumin levels found in the present study, after adjustments for various confounding factors, also were in accord with previous results in community-dwelling older adults from other countries. In a Japanese population of 80-year-old community residents, albumin levels are an independent predictor of all-cause mortality [34], and this result was also observed in 421 Japanese residents aged 69–71 years [35]. The same association was found in studies of community-dwelling Americans over 65 years of age [36–38]. However, the HRs obtained in this study were relatively smaller than those of the previous study. This may be related to the different characteristics of the age distribution of the study subjects, which were mostly concentrated below 80 years old in the previous study and none of them reached centennial age. To our knowledge, our study based on the considerable population-based centenarian samples to fill this gap in the field of centenarian research for the first time.

In addition, our findings may support the hypothesis that the association between hypoalbuminemia and the risk of death was enhanced with the increasing number of co-morbid CMDs. Notably, several studies on elderly patients with stroke or acute coronary syndrome observed low serum concentration of albumin is a negative prognostic index for in-hospital mortality [16, 22–24]. However, the above studies have focused exclusively on specific patients admitted to hospital, the short-term mortality is probably impacted by other factors during acute illness. For general older population, more recent data from 4947 persons participating in the Atherosclerosis Risk in Communities (ARIC) Study, showed an association of hypoalbuminemia with death, and this association was not modified by the presence of diabetes [38]. However, the association between hypoalbuminemia and death was weakened in the presence of diabetes in the Geisinger cohort study [38]. Therefore, research on the prognosis for the general participants with hypoalbuminemia combined with CMD is scant and inconsistent. Moreover, with the epidemic of multimorbidity, it is essential to explore whether the relationship between lower albumin levels and mortality was modified by the increasing number of CMDs. With the escalating burden of aging populations worldwide, multimorbidity is related to the reduced quality of life and the greater use of health-care resources, which is becoming a global health challenge [39]. An increasing prevalence of multimorbidity with age has been reported from 6.4 to 76.5% of elderly individuals in China [40]. Among these chronic diseases, CMDs, including hypertension, diabetes, CHD and stroke, are the main components of multimorbidity [41, 42]. In fact, 60.3% of the participants with hypoalbuminemia combined with at least single CMD, and a tendency of increasing the risk of mortality in our study. Therefore, this study expands upon previous studies and also highlights that the community-dwelling older adults with hypoalbuminemia with multiple co-existing CMDs should be given special attention and targeted for priority interventions to reduce the risk of adverse outcomes.

The antioxidant properties and the involvement in chronic inflammation of albumin may be able to partially explain the association between albumin level and the risk of all-cause mortality observed in this study. It was demonstrated that serum albumin exerts antioxidant activity by limiting reactive oxygen species (ROS) production via binding to ligands, such as metal ions and fatty acids, and by scavenging ROS through free radical-trapping activity [43]. Augmented albumin level at local inflammation sites have also been shown to have potent antioxidant activity [44]. This is supported by the inverse correlation between albumin levels and CRP levels in the present study. The reduced antioxidant and anti-inflammatory effects associated with hypoalbuminemia may increase the risk of death, especially in participants combined with multiple CMDs. A common point of the above CMDs is the appearance of oxidative stress and inflammation [45, 46], which further exacerbates the detrimental effect on mortality risk when the antioxidant and anti-inflammatory properties of albumin are also not fully exploited.

In addition, there is increasing evidence that among the older adults caloric–protein malnutrition is a widespread problem which is worsening with ongoing population ageing [47]. Albumin is increasingly being included by clinicians in attempted interventions as a sensitive indicator for nutritional screening [48]. Prior studies have shown that serum concentration of albumin is strongly correlated to the Mini Nutritional Assessment in older adults, and low albumin usually indicates the body’s malnutrition [49]. It has been reported that malnutrition is associated with significantly increased morbidity and mortality in independently living elderly, as well as the residents of nursing homes and hospitalized patients [50]. Moreover, evidence suggests that serum albumin levels may be associated with a wide range of cardiometabolic outcomes [51]. Additionally, as a key reaction to CMDs, insulin resistance significantly affects nutritional metabolism and status [52]. This implies that on one hand, malnutrition may cause adverse cardiometabolic outcomes, whereas on the other hand, CMDs may exacerbate the malnutrition. This is in line with our results demonstrating that comorbid malnutrition and CMDs impact all-cause mortality risk in a dose-dependent manner.

Several strengths of our study, as well as innovations, are as follows. Firstly, our study focused on the special populations of centenarians with a community-based and large sample size, filling this gap in our current knowledge. Secondly, we took the further step of considering the impact of the number of comorbid CMDs in addition to hypoalbuminemia only. However, several possible limitations of the present study warrant special consideration. Firstly, centenarians may be exposed to more factors affecting albumin levels, such as disability and chronic kidney disease. However, we conducted sensitivity analyses by excluding those with ADL disability or adding the adjustment of glomerular filtration rate and the results were materially unchanged. Secondly, survey data on medication taking in the questionnaire were inaccurate due to memory bias in older adults and there were relatively large amounts of missing data, so medication information was not included in the analysis. Thirdly, the follow up duration was relatively short. However, our study population was centenarians with a relatively high mortality rate (52.1%), indicating ample statistical power to demonstrate the above issues clearly.

## Conclusions

Lower levels of albumin were associated with increased risks of all-cause death in a cohort of centenarians, and this risk was enhanced when hypoalbuminemia combined with multiple CMDs. Our findings highlight that those with hypoalbuminemia and co-existing multiple CMDs deserves more attentions from community and clinicians to prevent adverse outcomes.

### Supplementary Information

**Additional file 1: Figure S1.** Distribution of serum albumin levels of the participants at baseline. **Figure S2.** Kaplan–Meier curves for all-cause death by quartiles of serum albumin levels. **Figure S3. **The Kaplan–Meier curves were used to compare the difference between the normoalbuminemia and hypoalbuminemia groups. **Figure S4.** HRs and 95%CI of risks of all-cause death associated with cardiometabolic diseases. Adjusted for sex, age, ethnicity, marriage, educational levels, smoking status, alcohol drinking status, hemoglobin, CRP, dyslipidemia and hypoalbuminemia. CI, confidence interval; CRP, C-reactive protein; HR, hazard ratio. **Table S1.** Baseline characteristics of the entire cohort of centenarians by survival status. **Table S2.** Risks of all-cause death associated with hypoalbuminemia combined with cardiometabolic diseases which included dyslipidemia. **Table S3.** Risks of all-cause death associated with hypoalbuminemia and hypoalbuminemia combined with cardiometabolic diseases after excluding patients with ADL disability. **Table S4.** Risks of all-cause death associated with hypoalbuminemia and hypoalbuminemia combined with cardiometabolic diseases additionally adjusted for eGFR.

## Data Availability

In an attempt to preserve the privacy of individuals, clinical data will not be shared. Data can be obtained from the corresponding author upon reasonable request.

## Abbreviations

ADL: Activities of daily living
ALT: Alanine transaminase
AST: Aspartate aminotransferase
CHCCS: China Hainan Centenarian Cohort Study
CHD: Coronary heart diseases
CMD: Cardiometabolic disease
CRP: C-reactive protein
eGFR: Estimated glomerular filtration rate
FBG: Fasting blood glucose
HDL-C: High density lipoprotein cholesterol
HR: Hazard ratio
LDL-C: Low density lipoprotein cholesterol
TC: Total cholesterol
TG: Triglyceride

## References

[1] Kuwahata M, Hasegawa M, Kobayashi Y, Wada Y, Kido Y (2017). An oxidized/reduced state of plasma albumin reflects malnutrition due to an insufficient diet in rats. J Clin Biochem Nutr.

[2] Zhang J, Ding Y, Wang W, Lu Y, Wang H, Wang H, Teng L (2020). Combining the fibrinogen/albumin ratio and systemic inflammation response index predicts survival in resectable gastric cancer. Gastroenterol Res Pract.

[3] Keller U (2019). Nutritional laboratory markers in malnutrition. J Clin Med.

[4] Akirov A, Gorshtein A, Adler-Cohen C, Steinmetz T, Shochat T, Shimon I (2020). Low serum albumin levels predict short- and long-term mortality risk in patients hospitalised to general surgery wards. Intern Med J.

[5] Wu CY, Hu HY, Huang N, Chou YC, Li CP, Chou YJ (2018). Albumin levels and cause-specific mortality in community-dwelling older adults. Prev Med.

[6] Jin X, Xiong S, Ju SY, Zeng Y, Yan LL, Yao Y (2020). Serum 25-Hydroxyvitamin D, albumin, and mortality among chinese older adults: a population-based longitudinal study. J Clin Endocrinol Metab.

[7] Cabrerizo S, Cuadras D, Gomez-Busto F, Artaza-Artabe I, Marín-Ciancas F, Malafarina V (2015). Serum albumin and health in older people: Review and meta analysis. Maturitas.

[8] Lai CC, You JF, Yeh CY, Chen JS, Tang R, Wang JY, Chin CC (2011). Low preoperative serum albumin in colon cancer: a risk factor for poor outcome. Int J Colorectal Dis.

[9] Kim KJ, Lee BW (2012). The roles of glycated albumin as intermediate glycation index and pathogenic protein. Diabetes Metab J.

[10] Sitar ME, Aydin S, Cakatay U (2013). Human serum albumin and its relation with oxidative stress. Clin Lab.

[11] Roche M, Rondeau P, Singh NR, Tarnus E, Bourdon E (2008). The antioxidant properties of serum albumin. FEBS Lett.

[12] Gabay C, Kushner I (1999). Acute-phase proteins and other systemic responses to inflammation. N Engl J Med.

[13] Bologa RM, Levine DM, Parker TS, Cheigh JS, Serur D, Stenzel KH, Rubin AL (1998). Interleukin-6 predicts hypoalbuminemia, hypocholesterolemia, and mortality in hemodialysis patients. Am J Kidney Dis.

[14] Danesh J, Muir J, Wong YK, Ward M, Gallimore JR, Pepys MB (1999). Risk factors for coronary heart disease and acute-phase proteins A population-based study. Eur Heart J.

[15] Lis CG, Grutsch JF, Vashi PG, Lammersfeld CA (2003). Is serum albumin an independent predictor of survival in patients with breast cancer?. JPEN J Parenter Enteral Nutr.

[16] González-Pacheco H, Amezcua-Guerra LM, Sandoval J, Martínez-Sánchez C, Ortiz-León XA, Peña-Cabral MA, Bojalil R (2017). Prognostic implications of serum albumin levels in patients with acute coronary syndromes. Am J Cardiol.

[17] Sun J, Su H, Lou Y, Wang M (2021). Association between serum albumin level and all-cause mortality in patients with chronic kidney disease: a retrospective cohort study. Am J Med Sci.

[18] Djoussé L, Rothman KJ, Cupples LA, Levy D, Ellison RC (2002). Serum albumin and risk of myocardial infarction and all-cause mortality in the Framingham Offspring Study. Circulation.

[19] Lyall DM, Celis-Morales CA, Anderson J, Gill JM, Mackay DF, McIntosh AM, Smith DJ, Deary IJ, Sattar N, Pell JP (2017). Associations between single and multiple cardiometabolic diseases and cognitive abilities in 474 129 UK Biobank participants. Eur Heart J.

[20] Wang Z, Marseglia A, Shang Y, Dintica C, Patrone C, Xu W (2020). Leisure activity and social integration mitigate the risk of dementia related to cardiometabolic diseases: a population-based longitudinal study. Alzheimers Dement.

[21] Ma T, He L, Luo Y, Zhang G, Cheng X, Bai Y (2022). Use of fish oil and mortality of patients with cardiometabolic multimorbidity: a prospective study of UK biobank. Nutr Metab Cardiovasc Dis.

[22] Arques S, Roux E, Sbragia P, Gelisse R, Pieri B, Ambrosi P (2008). Usefulness of serum albumin concentration for in-hospital risk stratification in frail, elderly patients with acute heart failure Insights from a prospective, monocenter study. Int J Cardiol.

[23] Famakin B, Weiss P, Hertzberg V, McClellan W, Presley R, Krompf K, Karp H, Frankel MR (2010). Hypoalbuminemia predicts acute stroke mortality: paul coverdell georgia stroke registry. J Stroke Cerebrovasc Dis.

[24] Gariballa SE (2001). Malnutrition in hospitalized elderly patients: when does it matter?. Clin Nutr.

[25] Carter AM, Catto AJ, Mansfield MW, Bamford JM, Grant PJ (2007). Predictive variables for mortality after acute ischemic stroke. Stroke.

[26] He Y, Zhao Y, Yao Y, Yang S, Li J, Liu M, Chen X, Wang J, Zhu Q, Li X (2018). Cohort profile: The China Hainan Centenarian Cohort Study (CHCCS). Int J Epidemiol.

[27] Gatta A, Verardo A, Bolognesi M (2012). Hypoalbuminemia. Intern Emerg Med.

[28] Whelton PK, Carey RM, Aronow WS, Casey DE, Collins KJ, Dennison Himmelfarb C, DePalma SM, Gidding S, Jamerson KA, Jones DW (2018). 2017 ACC/AHA/AAPA/ABC/ACPM/AGS/APhA/ASH/ASPC/NMA/PCNA guideline for the prevention, detection, evaluation, and management of high blood pressure in adults: a report of the american college of cardiology/american heart association task force on clinical practice guidelines. Hypertension.

[29] Schwarz PE, Gruhl U, Bornstein SR, Landgraf R, Hall M, Tuomilehto J (2007). The European perspective on diabetes prevention: development and implementation of A European Guideline and training standards for diabetes prevention (IMAGE). Diab Vasc Dis Res.

[30] Joint Committee on Revision of guidelines for prevention and treatment of dyslipidemia in Chinese adults Guidelines for the prevention and treatment of dyslipidemia in Chinese adults (revised in 2016). Chin Circul J 2016, 31(10):937–950.

[31] Mahoney FI, Barthel DW (1965). Functional evaluation: the barthel index. Md State Med J.

[32] Levey AS, Stevens LA, Schmid CH, Zhang YL, Castro AF, Feldman HI, Kusek JW, Eggers P, Van Lente F, Greene T (2009). A new equation to estimate glomerular filtration rate. Ann Intern Med.

[33] Ortiz R, Kluwe B, Lazarus S, Teruel MN, Joseph JJ (2022). Cortisol and cardiometabolic disease: a target for advancing health equity. Trends Endocrinol Metab.

[34] Takata Y, Ansai T, Soh I, Awano S, Sonoki K, Akifusa S, Kagiyama S, Hamasaki T, Torisu T, Yoshida A (2010). Serum albumin levels as an independent predictor of 4-year mortality in a community-dwelling 80-year-old population. Aging Clin Exp Res.

[35] Shibata H, Haga H, Nagai H, Suyama Y, Yasumura S, Koyano W, Suzuki T (1992). Predictors of all-cause mortality between ages 70 and 80: the Koganei study. Arch Gerontol Geriatr.

[36] Fried LP, Kronmal RA, Newman AB, Bild DE, Mittelmark MB, Polak JF, Robbins JA, Gardin JM (1998). Risk factors for 5-year mortality in older adults: the Cardiovascular Health Study. JAMA.

[37] Corti MC, Guralnik JM, Salive ME, Sorkin JD (1994). Serum albumin level and physical disability as predictors of mortality in older persons. JAMA.

[38] Shannon CM, Ballew SH, Daya N, Zhou L, Chang AR, Sang Y, Coresh J, Selvin E, Grams ME (2021). Serum albumin and risks of hospitalization and death: findings from the atherosclerosis risk in communities study. J Am Geriatr Soc.

[39] Skou ST, Mair FS, Fortin M, Guthrie B, Nunes BP, Miranda JJ, Boyd CM, Pati S, Mtenga S, Smith SM (2022). Multimorbidity. Nat Rev Dis Primers.

[40] Yao SS, Cao GY, Han L, Chen ZS, Huang ZT, Gong P, Hu Y, Xu B (2020). Prevalence and patterns of multimorbidity in a nationally representative sample of older chinese: results from the china health and retirement longitudinal study. J Gerontol A Biol Sci Med Sci.

[41] Hu X, Huang J, Lv Y, Li G, Peng X (2015). Status of prevalence study on multimorbidity of chronic disease in China: systematic review. Geriatr Gerontol Int.

[42] Busija L, Lim K, Szoeke C, Sanders KM, McCabe MP (2019). Do replicable profiles of multimorbidity exist? systematic review and synthesis. Eur J Epidemiol.

[43] Suriyaprom K, Kaewprasert S, Putpadungwipon P, Namjuntra P, Klongthalay S (2019). Association of antioxidant status and inflammatory markers with metabolic syndrome in Thais. J Health Popul Nutr.

[44] Jin SM, Hong YJ, Jee JH, Bae JC, Hur KY, Lee MK, Kim JH (2016). Change in serum albumin concentration is inversely and independently associated with risk of incident metabolic syndrome. Metabolism.

[45] Brouwer-Brolsma EM, van Lee L, Streppel MT, Sluik D, van de Wiel AM, de Vries JHM, Geelen A, Feskens EJM (2018). Nutrition questionnaires plus (NQplus) study, a prospective study on dietary determinants and cardiometabolic health in Dutch adults. BMJ Open.

[46] Parisi L, Gini E, Baci D, Tremolati M, Fanuli M, Bassani B, Farronato G, Bruno A, Mortara L (2018). Macrophage polarization in chronic inflammatory diseases: killers or builders?. J Immunol Res.

[47] Agarwal E, Miller M, Yaxley A, Isenring E (2013). Malnutrition in the elderly: a narrative review. Maturitas.

[48] Wang CY, Fu PK, Chao WC, Wang WN, Chen CH, Huang YC (2020). Full versus trophic feeds in critically ill adults with high and low nutritional risk scores: a randomized controlled trial. Nutrients.

[49] Zhang Z, Pereira SL, Luo M, Matheson EM (2017). Evaluation of blood biomarkers associated with risk of malnutrition in older adults: a systematic review and meta-analysis. Nutrients.

[50] Hickson M (2006). Malnutrition and ageing. Postgrad Med J.

[51] Seidu S, Kunutsor SK, Khunti K (2020). Serum albumin, cardiometabolic and other adverse outcomes: systematic review and meta-analyses of 48 published observational cohort studies involving 1,492,237 participants. Scand Cardiovasc J.

[52] Feng L, Chu Z, Quan X, Zhang Y, Yuan W, Yao Y, Zhao Y, Fu S (2022). Malnutrition is positively associated with cognitive decline in centenarians and oldest-old adults: a cross-sectional study. EClinicalMedicine.

