# Supplementary material for: Multiple cardiometabolic diseases enhance the adverse effects of hypoalbuminemia on mortality among centenarians in China: a cohort study
Source: Diabetol Metab Syndr. 2023 Nov 14;15:231. doi: 10.1186/s13098-023-01201-y (PMC10644513; doi:10.1186/s13098-023-01201-y)
Supplement: Supplementary file 1 — Additional file 1: Figure S1. Distribution of serum albumin levels of the participants at baseline. Figure S2. Kaplan–Meier curves for all-cause death by quartiles of serum albumin levels. Figure S3. The Kaplan–Meier curves were used to compare the difference between the normoalbuminemia and hypoalbuminemia groups. Figure S4. HRs and 95%CI of risks of all-cause death associated with cardiometabolic diseases. Adjusted for sex, age, ethnicity, marriage, educational levels, smoking status, alcohol drinking status, hemoglobin, CRP, dyslipidemia and hypoalbuminemia. CI, confidence interval; CRP, C-reactive protein; HR, hazard ratio. Table S1. Baseline characteristics of the entire cohort of centenarians by survival status. Table S2. Risks of all-cause death associated with hypoalbuminemia combined with cardiometabolic diseases which included dyslipidemia. Table S3. Risks of all-cause death associated with hypoalbuminemia and hypoalbuminemia combined with cardiometabolic diseases after excluding patients with ADL disability. Table S4. Risks of all-cause death associated with hypoalbuminemia and hypoalbuminemia combined with cardiometabolic diseases additionally adjusted for eGFR. [file 13098_2023_1201_MOESM1_ESM.docx]

**Additional file**

**
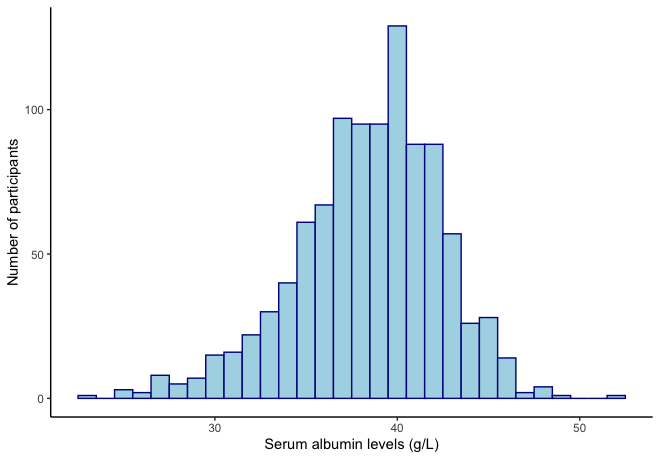
**

Additional file Figure 1. Distribution of serum albumin levels of the participants at baseline.


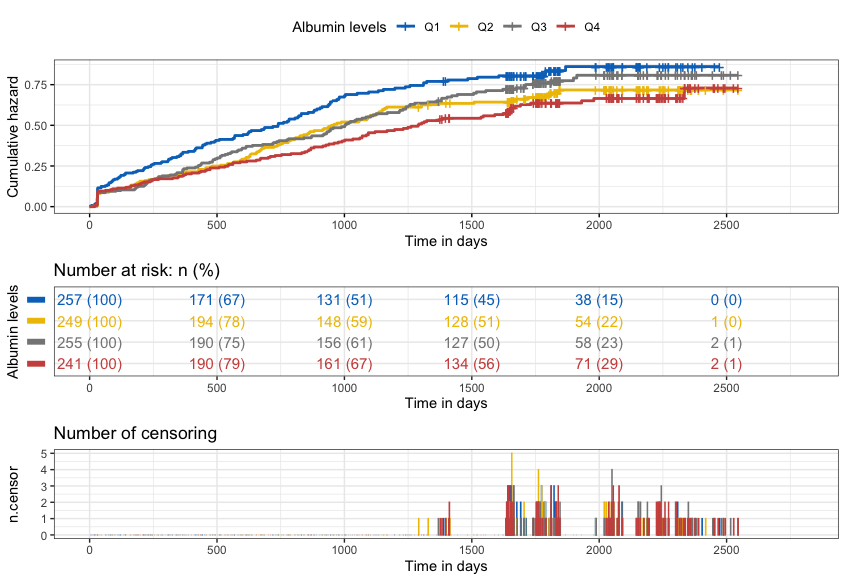


Additional file Figure 2. Kaplan-Meier curves for all-cause death by quartiles of serum albumin levels.


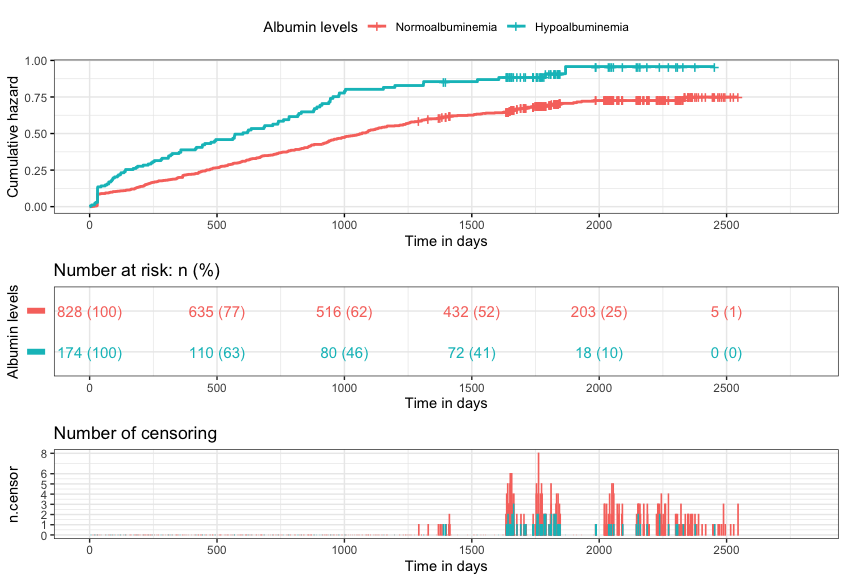


Additional file Figure 3.The Kaplan-Meier curves were used to compare the difference between the normoalbuminemia and hypoalbuminemia groups.


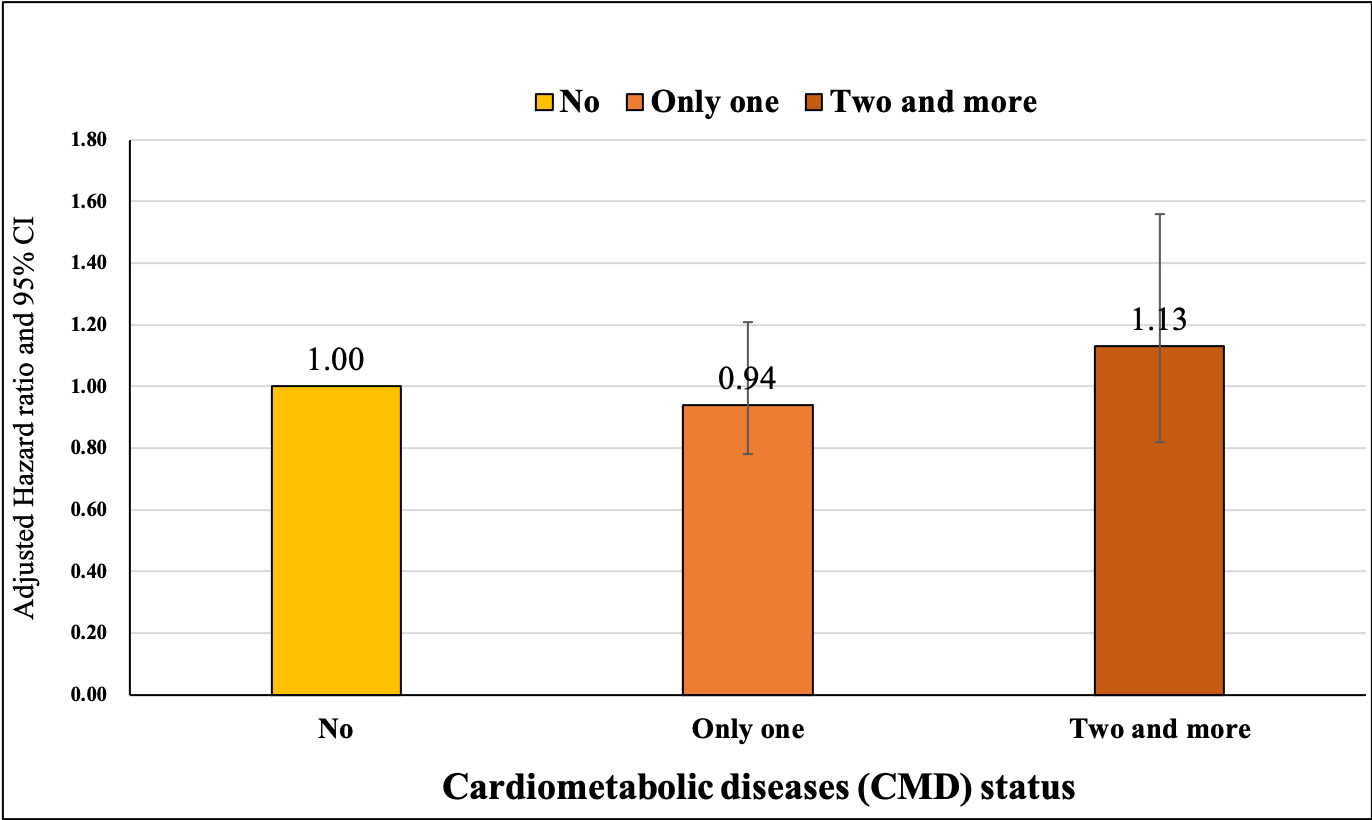


Additional file Figure 4. HRs and 95%CI of risks of all-cause death associated with cardiometabolic diseases.

Adjusted for sex, age, ethnicity, marriage, educational levels, smoking status, alcohol drinking status, hemoglobin, CRP, dyslipidemia and hypoalbuminemia.

CI, confidence interval; CRP, C-reactive protein; HR, hazard ratio.

Additional file Table 1. Baseline characteristics of the entire cohort of centenarians by survival status.

|  | Total | Serum albumin | | *P*value |
| --- | --- | --- | --- | --- |
|  |  | Alive Throughout the Study | Dead during the Study |  |
| Age(year), median(IQR) | 102(101-104) | 102(101-104) | 102(101-104) | 0.406 |
| Female, n(%) | 822(82.0) | 400(83.3) | 422(80.8) | 0.305 |
| Han ethnicity, n(%) | 883(88.1) | 427(89.0) | 456(87.4) | 0.434 |
| Widowed, n(%) | 836(83.4) | 394(82.1) | 442(84.7) | 0.270 |
| Illiterate, n(%) | 915(91.3) | 438(91.3) | 477(91.4) | 0.943 |
| Never smoking, n(%) | 896(89.4) | 443(92.3) | 453(86.8) | 0.005 |
| Never alcohol drinking, n(%) | 836(83.4) | 402(83.8) | 434(83.1) | 0.796 |
| ADL disability, n(%) | 140(14.0) | 48(10.0) | 92(17.6) | 0.001 |
| Hypertension, n(%) | 744(74.3) | 351(73.1) | 393(75.3) | 0.434 |
| Diabetes, n(%) | 96(9.6) | 55(11.5) | 41(7.9) | 0.053 |
| CHD, n(%) | 41(4.1) | 18(3.8) | 23(4.4) | 0.600 |
| Stroke, n(%) | 22(2.2) | 9(1.9) | 13(2.5) | 0.507 |
| Dyslipidemia, n(%) | 230(23.0) | 123(25.6) | 107(20.5) | 0.054 |
| BMI(kg/m^2^ ), mean±SD | 18.04±3.41 | 18.20±3.19 | 17.73±3.63 | 0.002 |
| SBP(mmHg), mean±SD | 152.57±24.43 | 153.17±24.14 | 152.01±24.70 | 0.453 |
| DBP(mmHg), mean±SD | 75.75±12.94 | 75.35±12.28 | 76.11±13.52 | 0.353 |
| CRP (mg/L), median(IQR) | 0.19(0.08-0.43) | 0.19(0.07-0.35) | 0.19(0.08-0.55) | 0.772 |
| Haemoglobin (g/L), mean±SD | 113.05±16.35 | 112.79±15.84 | 113.13±17.40 | 0.735 |
| FBG(mmol/L), median(IQR) | 4.82(4.26-5.64) | 4.82(4.24-5.68) | 4.82(4.27-5.60) | 0.819 |
| eGFR(mL/min/1.73 m^2^), median(IQR) | 76.42  (48.62-100.96) | 76.41(47.39-101.49) | 76.42(49.57-100.69) | 0.656 |
| Total protein (g/L), mean±SD | 68.65±6.27 | 68.42±5.69 | 68.86±6.77 | 0.269 |
| ALT (UI/L), median(IQR) | 9.20(7.20-12.00) | 9.30(7.30-11.73) | 9.0(7.10-12.35) | 0.869 |
| AST (UI/L), median(IQR) | 20.60(17.50-24.40) | 20.60(17.50-24.20) | 20.6(17.45-24.50) | 0.924 |
| Serum albumin (g/L), mean±SD | 38.49±4.00 | 38.74±3.68 | 38.13±4.39 | 0.028 |
| Hypoalbuminemia, n(%) | 174(17.4) | 70(14.6) | 104(19.9) | 0.026 |

ADL, activities of daily living; ALT, alanine transaminase; AST, aspartate aminotransferase; BMI, body mass index; CHD, coronary heart diseases; CRP, C-reactive protein; DBP, diastolic blood pressure; eGFR, estimated glomerular filtration rate; FBG, fasting blood glucose; HDL, high-density lipoprotein; IQR, interquartile ranges; LDL, low-density lipoprotein; SBP, systolic blood pressure; SD, standard deviation; TC, total cholesterol; TG, triglycerides.

Additional file Table 2. Risks of all-cause death associated with hypoalbuminemia combined with cardiometabolic diseases which included dyslipidemia.

| Adverse outcome | Normoalbuminemia | Hypoalbuminemia combined with cardiometabolic diseases which included dyslipidemia | | | |
| --- | --- | --- | --- | --- | --- |
|  |  | 0 | 1 | ≥ 2 | *P* for trend |
| Mortality | 418(50.5%) | 21(60.0%) | 56(59.6%) | 27(60.0%) | 0.152 |
| HR (95% CI) | | | | | |
| Model 1 | 1 | 1.56(1.01-2.42) | 1.34(1.01-1.77) | 1.46(0.99-2.16) | 0.004 |
| Model 2 | 1 | 1.59(1.02-2.48) | 1.34(1.01-1.77) | 1.45(0.98-2.14) | 0.004 |
| Model 3 | 1 | 1.54(0.99-2.41) | 1.36(1.02-1.79) | 1.51(1.02-2.23) | 0.002 |
| Model 4 | 1 | 1.60(1.01-2.52) | 1.40(1.04-1.88) | 1.56(1.04-2.34) | 0.003 |

CI, confidence interval; CRP, C-reactive protein; HR, hazard ratio.

Model 1: No adjustment for any covariates.

Model 2: Adjusted for sex, age, ethnicity, marriage, educational levels.

Model 3: Model 2 plus smoking status, alcohol drinking status.

Model 4: Model 3 plus hemoglobin, CRP.

Additional file Table 3. Risks of all-cause death associated with hypoalbuminemia and hypoalbuminemia combined with cardiometabolic diseases after excluding patients with ADL disability.

| Adverse outcome | Normoalbuminemia | Hypoalbuminemia* | Hypoalbuminemia combined with cardiometabolic diseases | | |  |
| --- | --- | --- | --- | --- | --- | --- |
|  |  |  | 0 | 1 | ≥ 2 | *P* for trend |
| Mortality | 365(48.9%) | 65(56.5) | 21(56.8%) | 37(55.2%) | 7(63.6%) | 0.456 |
| HR (95% CI) | | | | | | |
| Model 1 | 1 | 1.32(1.02-1.72) | 1.41(0.91-2.19) | 1.22(0.87-1.71) | 1.83(0.87-3.87) | 0.044 |
| Model 2 | 1 | 1.33(1.02-1.74) | 1.43(0.91-2.25) | 1.22(0.87-1.71) | 1.99(0.93-4.25) | 0.040 |
| Model 3 | 1 | 1.42(1.09-1.87) | 1.44(0.91-2.26) | 1.28(0.91-1.81) | 2.12(0.99-4.53) | 0.017 |
| Model 4 | 1 | 1.49(1.12-1.99) | 1.51(0.95-2.40) | 1.36(0.95-1.94) | 2.22(1.03-4.78) | 0.009 |

CHD, coronary heart diseases; ADL, activities of daily living; CI, confidence interval; CRP, C-reactive protein; HR, hazard ratio.

Model 1: No adjustment for any covariates.

Model 2: Adjusted for sex, age, ethnicity, marriage, educational levels.

Model 3: Model 2 plus smoking status, alcohol drinking status, dyslipidemia.

Model 4: Model 3 plus hemoglobin, CRP.

* Model 4 was additionally adjusted for hypertension, diabetes, CHD and stroke.

| Adverse outcome | Normoalbuminemia | Hypoalbuminemia* | Hypoalbuminemia combined with cardiometabolic diseases | | | | |  |
| --- | --- | --- | --- | --- | --- | --- | --- | --- |
|  |  |  | 0 | | 1 | | ≥ 2 | *P* for trend |
| Mortality | 418(50.5%) | 104(59.8%) | 32(59.4%) | | 62(59.0%) | | 10(66.7%) | 0.152 |
| HR (95% CI) |  | | |  |  |  |  |  |
| Model 1 | 1 | 1.41(1.14-1.75) | 1.44(1.01-2.06) | | 1.34(1.03-1.75) | | 1.86(0.99-3.48) | 0.002 |
| Model 2 | 1 | 1.41 (1.14-1.75) | 1.44(1.00-2.08) | | 1.34(1.02-1.75) | | 1.97(1.04-3.71) | 0.002 |
| Model 3 | 1 | 1.43 (1.15-1.78) | 1.48(1.02-2.13) | | 1.39(1.06-1.81) | | 2.09(1.11-3.93) | 0.001 |
| Model 4 | 1 | 1.56 (1.22-1.98) | 1.55(1.06-2.27) | | 1.45(1.09-1.93) | | 2.15(1.14-4.08) | 0.001 |

Additional file Table 4. Risks of all-cause death associated with hypoalbuminemia and hypoalbuminemia combined with cardiometabolic diseases additionally adjusted for eGFR.

CHD, coronary heart diseases; CI, confidence interval; CRP, C-reactive protein; eGFR, estimated glomerular filtration rate; HR, hazard ratio.

Model 1: No adjustment for any covariates.

Model 2: Adjusted for sex, age, ethnicity, marriage, educational levels.

Model 3: Model 2 plus smoking status, alcohol drinking status, dyslipidemia.

Model 4: Model 3 plus hemoglobin, CRP and eGFR.

* Model 4 was additionally adjusted for hypertension, diabetes, CVD and stroke.
